# Supplementary figures and images for: Monitoring and analysis of the expansion of the Ajmr Port, Davao City, Philippines using multi-source remote sensing data
Source: PeerJ. 2019 Aug 19;7:e7512. doi: 10.7717/peerj.7512 (PMC6705383; doi:10.7717/peerj.7512)

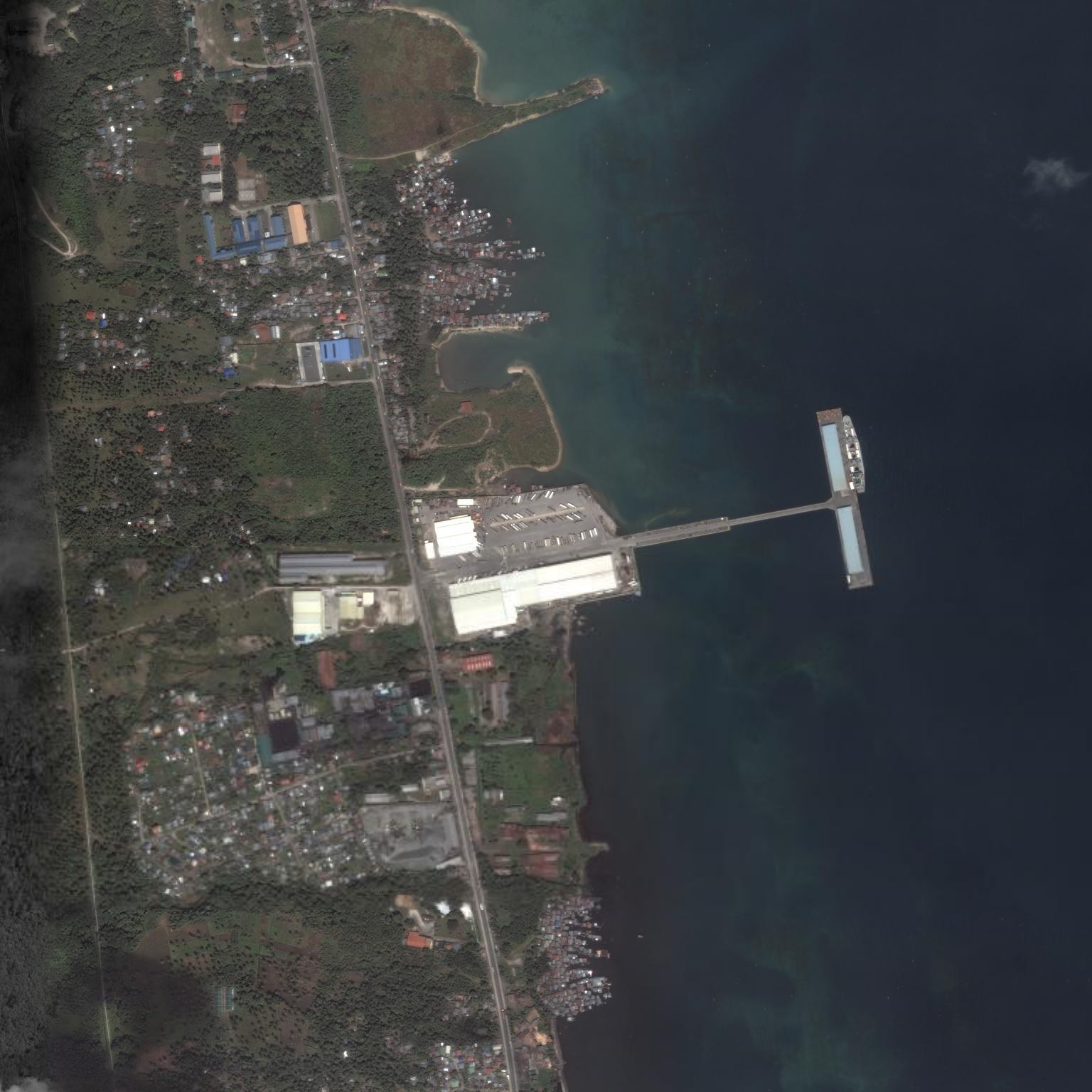

Supplement: Supplemental Information 1 — Google Earth High Definition imagery [file peerj-07-7512-s001.tif]

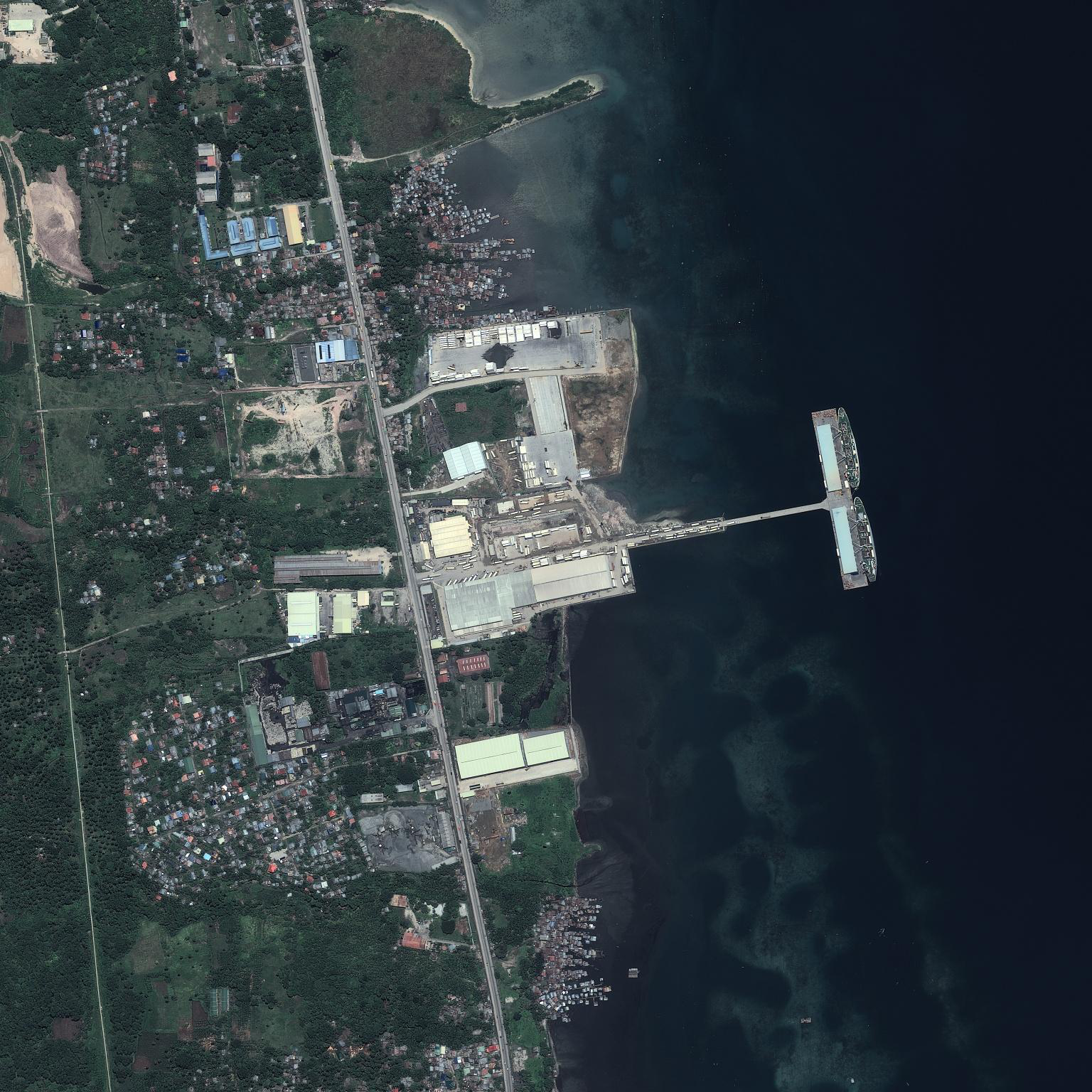

Supplement: Supplemental Information 2 — Google Earth High Definition imagery [file peerj-07-7512-s002.tif]

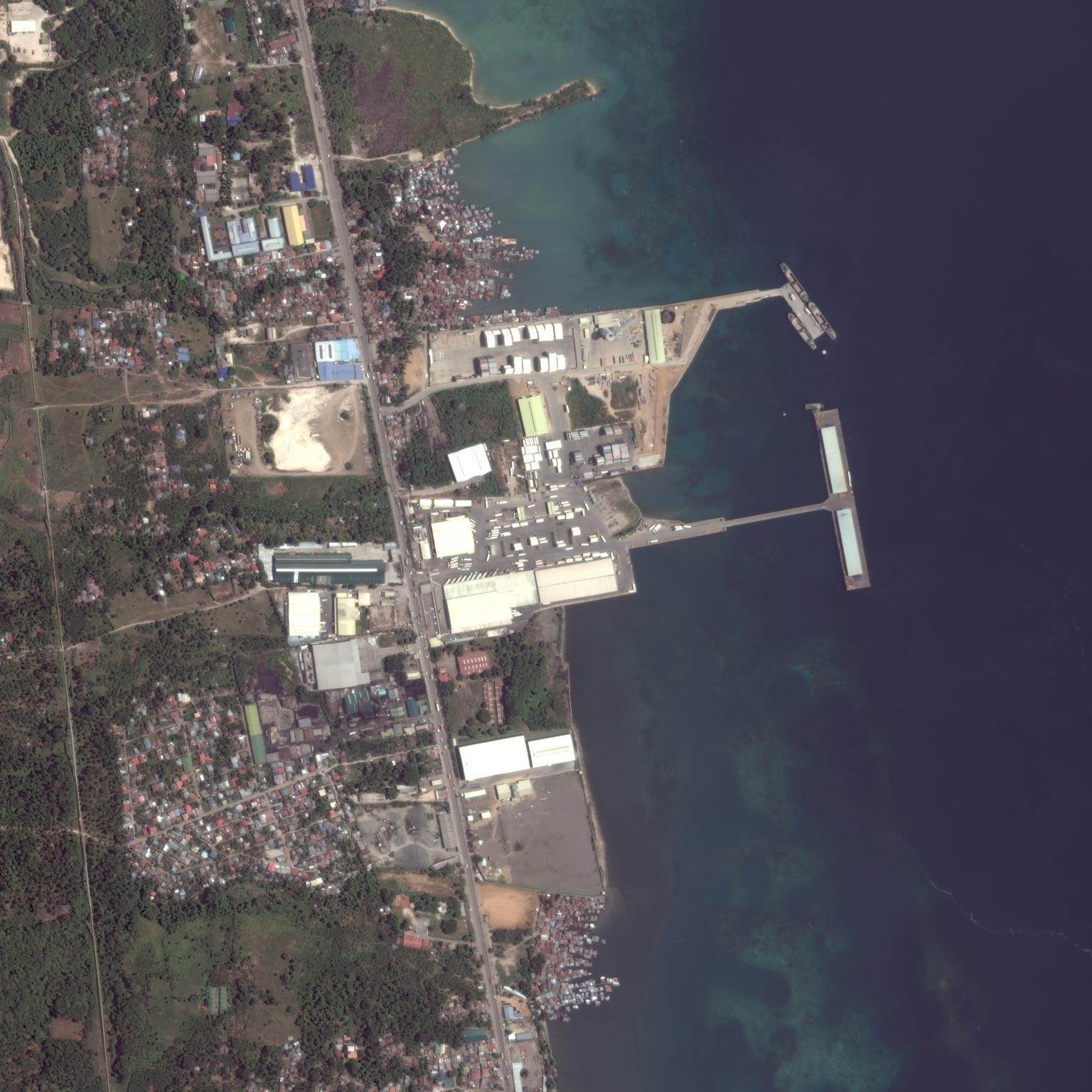

Supplement: Supplemental Information 3 — Google Earth High Definition imagery [file peerj-07-7512-s003.tif]

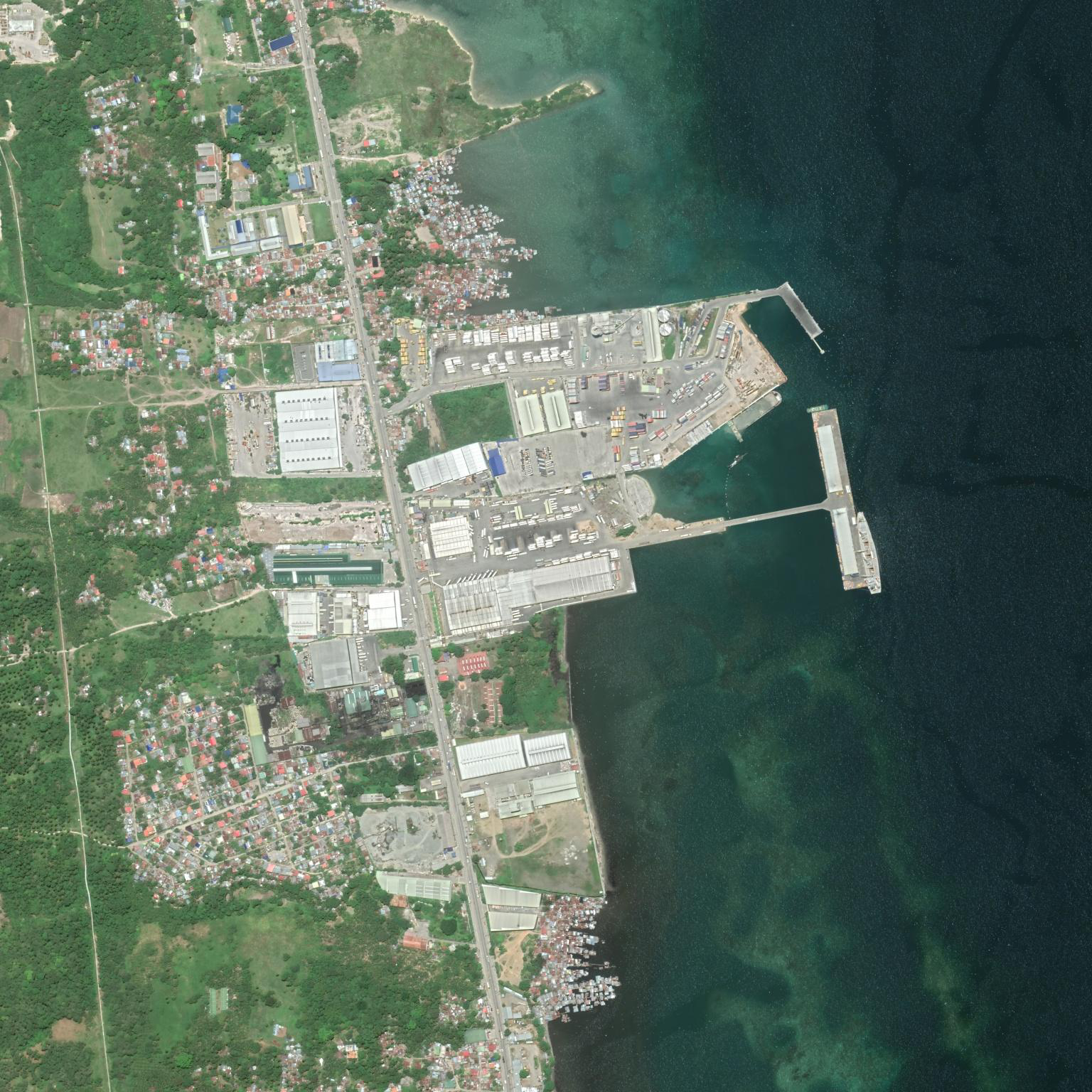

Supplement: Supplemental Information 4 — Google Earth High Definition imagery [file peerj-07-7512-s004.tif]
